# Dataset of target mass spectromic proteome profiling for human chromosome 18

**DOI:** 10.1016/j.dib.2016.07.034

**Published:** 2016-07-26

**Authors:** Ekaterina V. Ilgisonis, Arthur T. Kopylov, Victor G. Zgoda

**Affiliations:** Orekhovich Institute of Biomedical Chemistry, Moscow, Russia

## Abstract

Proteome profiling is a type of quantitative analysis that reveals level of protein expression in the sample. Proteome profiling by using selected reaction monitoring is an approach for the Chromosome-centric Human Proteome Project (C-HPP). Here we describe dataset generated in the course of the pilot phase of Russian part of C-HPP, which was focused on human Chr 18 proteins. Proteome profiling was performed using stable isotope-labeled standards (SRM/SIS) for plasma, liver tissue and HepG2 cells. Dataset includes both positive and negative results of protein detection.

These data were partly discussed in recent publications, “Chromosome 18 Transcriptome Profiling and Targeted Proteome Mapping in Depleted Plasma, Liver Tissue and HepG2 Cells” [1] and “Chromosome 18 transcriptoproteome of liver tissue and HepG2 Cells and targeted proteome mapping in depleted plasma: Update 2013” [2], supporting the accompanying publication “State of the Chromosome 18-centric HPP in 2016: Transcriptome and Proteome Profiling of Liver Tissue and HepG2 Cells” [3], and are deposited at the ProteomeXchange via the PASSEL repository with the dataset identifier PASSEL: PASS00697 for liver and HepG2 cell line.

**Specifications Table**

**Table:** 

Subject area	Biology
More specific subject area	Targeted mass-spectrometric proteome profiling of liver and HepG2 cell line
Type of data	Figure, table, raw files (.d), skyline files (.sky)
How data was acquired	Proteome profiling was performed using stable isotope-labeled standards (SRM/SIS) for liver tissue and HepG2 cells
Data format	Raw
Experimental factors	The trypsin digestion was used.
Experimental features	Digested samples were separated using the HPLC Agilent 1290 system including pump and autosampler. Internal Standard were produced using Overture (Protein Technologies, USA) or Hamilton Microlab STAR devices. The quantitative SRM analysis was performed using Agilent 6495 Triple Quadrupole (Agilent, USA) equipped with Jet Stream ionization source.
Data source location	Institute of Biomedical Chemistry, Moscow, Russia
Data accessibility	Data is available within this article and at the ProteomeXchange via PASSEL (http://proteomecentral.proteomexchange.org/cgi/GetDataset?ID=PXD004407).

**Value of the data**

• This data characterizes the diversity of chromosome 18 protein species in liver tissue and HepG2 cell line using SRM.
• This data could be of interest to laboratories studying protein reference levels and cross-tissue biological variability of proteome.
• This data could be useful for protein, peptide and transition selection for SRM-assay development.
• Dataset may be used as a test for automated SRM-data processing algorithms.

## 1 Data

This dataset describes conditions of liver tissue and HepG2 cell line proteome profiling. Targeted protein list included 268 proteins of chromosome 18. Data were automatically processed to quantify proteins in the biosample. Dataset includes raw data, transition list, skyline files and sample preparation instructions, available in PASSEL, 2 figures and Supplementary table with protein copy numbers in liver tissue and HepG2 cell line.

## 2 Experimental design, materials and methods

### 2.1 Sample preparation

The trypsin digestion of liver tissue and cell lysates was performed as described in Ponomarenko et al. [2].

### 2.2 Peptide synthesis

The peptides were produced using the SOLiD-phase peptide synthesis on the Overture (Protein Technologies, USA) or Hamilton Microlab STAR devices according to the method published in Hood et al. [4] . The isotope-labeled leucine (Fmoc–Leu–OH-13C6,15N) was used for isotope-labeled peptide synthesis instead of the unlabeled leucine (Fmoc–Leu–OH) [5].

### 2.3 Transition list

List of peptides for 268 chromosome 18 proteins was generated manually using data about occurrence of proteotypic peptides from proteomic repositories GPMdb, ProteinAtlas and PRIDE and MaRiMba-criteria (protocol was described earlier in Supplementary note 2, Zgoda et al. [1]). For each protein one “best-flyer” peptide was chosen. For each peptide 3 the most intensive transitions [6] were chosen using previous research results.

All 268 peptides were distributed over 3 SRM-assays (A–C) in equal parts according to their calculated retention time to avoid interference.

*LC-SRM Analysis* was held as described earlier in Supplementary note 2, Zgoda et al. [1]. Each SRM experiment was repeated in 3 technical runs. Each transition peak was characterized with the following variables: retention time, peak height, SIS/endogenous peak area ratio. No manual inspection for to find transitions that were similar to those in the target peptides or to reveal detected peptides was held.

### 2.4 How to use data

**Fig. 1:**
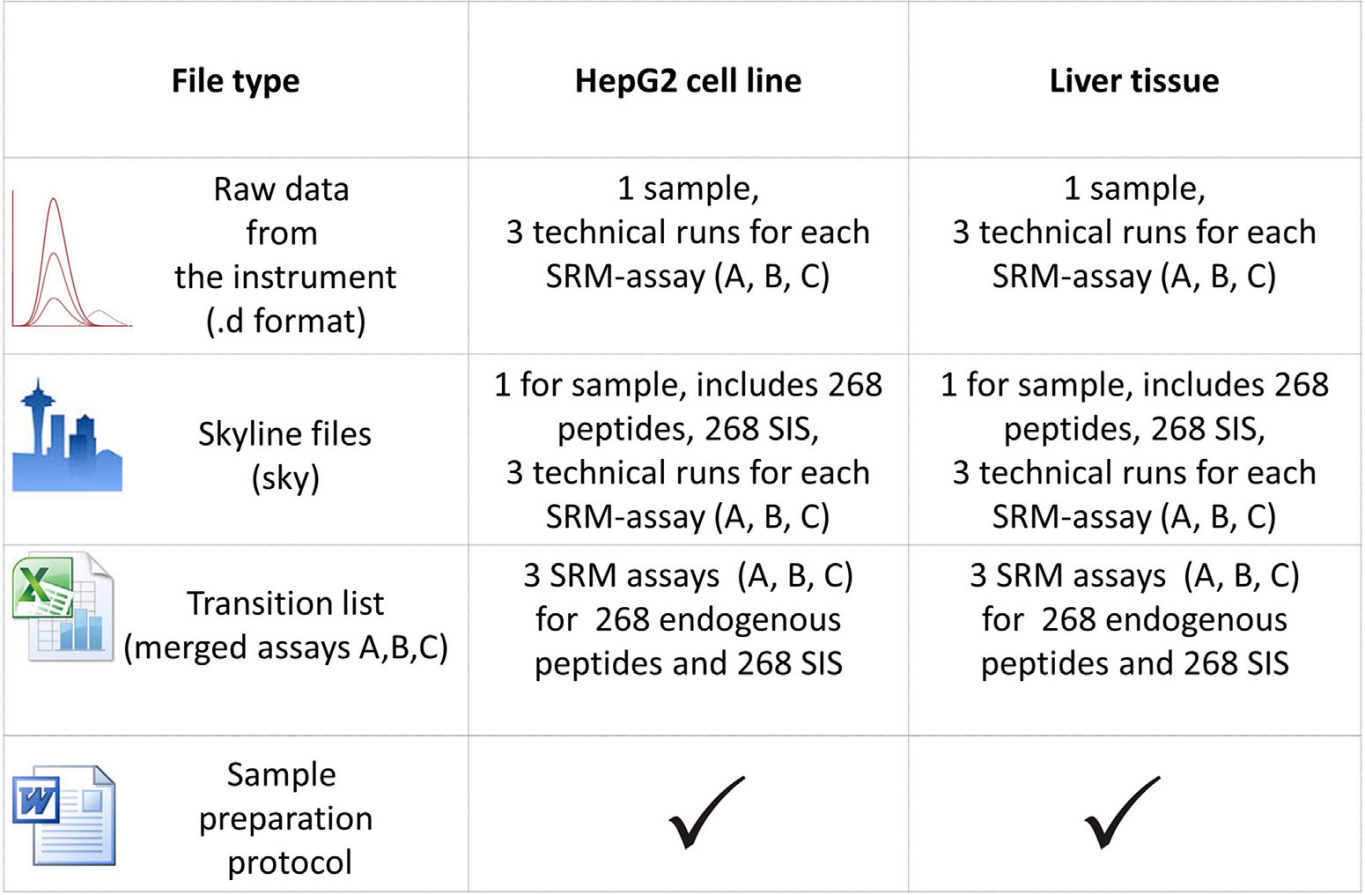
Chromosome 18 proteome profiling dataset scheme.

**Fig. 2:**
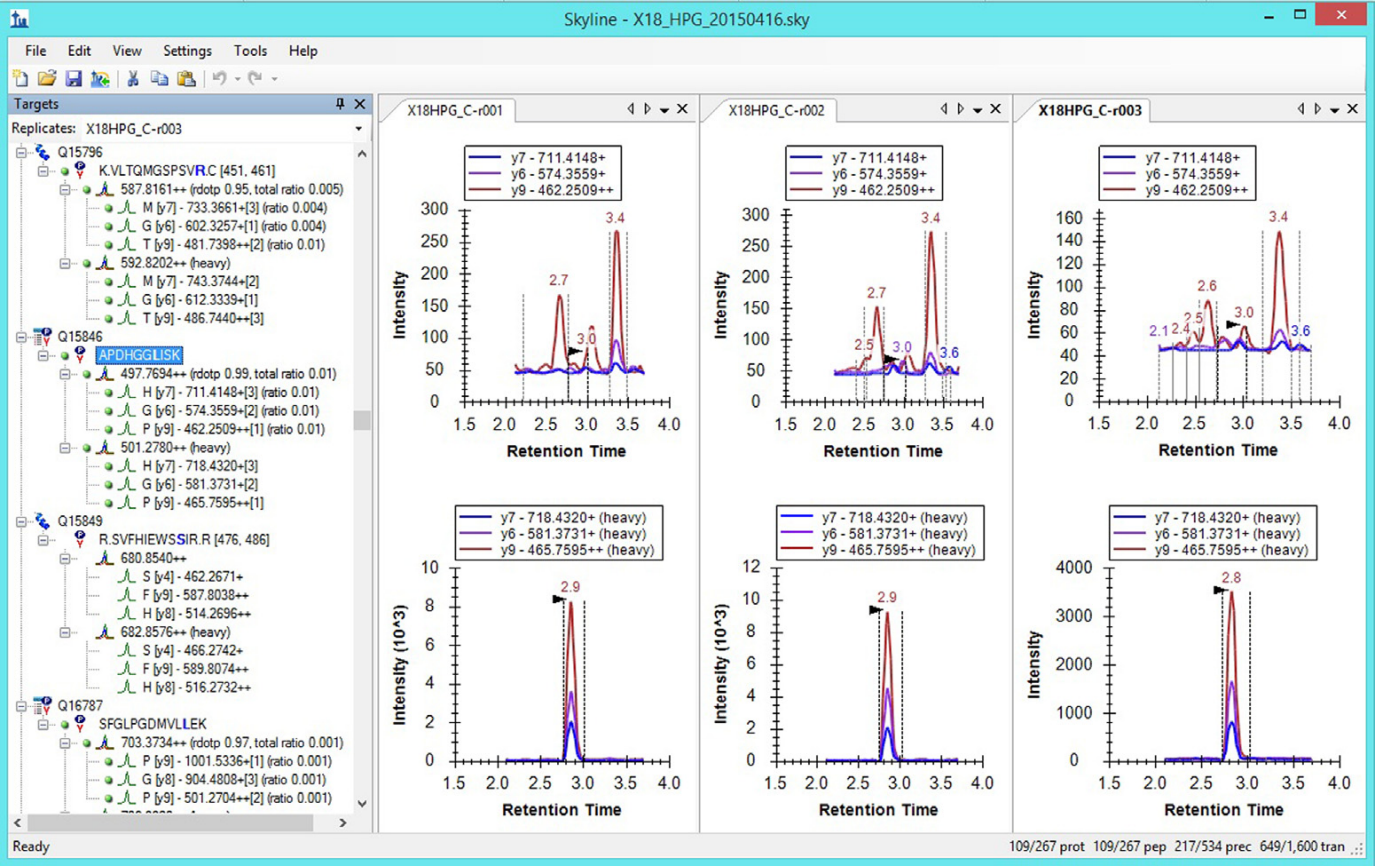
Screenshot of raw data visualization for HepG2 cell line, SRM-assay C using Skyline.

Dataset is represented by several file types (Fig. 1). For re-using of the dataset and extraction relevant information from it one can install freely-available and open source Windows client application Skyline [7] (for each biosample there is one skyline file, including transitions and technical runs info). It provides an opportunity to open raw data, visualize (Fig. 2) and analyze SRM data. Besides it is possible to use proprietary software (Agilent MassHunter Workstation Software) for data visualization.

All raw file are named using the following template:

1) Chromosome number: *X18;*
2) Sample type : *HLV* (liver tissue) or *HPG* (HepG2 cell line);
3) SRM-assay id: *A, B* or *C*; and
4) Technical run number: *roo1, r002, r003.*

For example, raw file name *X18HPG_C-r002.d* (Fig. 2). Skyline files also are named using these keys.

### 2.5 Quantification

Calibration curves were obtained for each of the desired peptides using the mixtures of purified synthetic non-labeled peptides in the concentration range of 100–100 fmole/µl and its isotopically labeled standards (SIS) were added at the concentration of 2 fmole/µl. All calibration curves were linear in the range of 100–0.1 fmole/µl and showed the coefficient of linear regression equal to 0.95.

Prior to the sample processing, the performance of the LC-SRM platforms used was validated by obtaining the calibration curves of the corresponding set of SIS and synthetic non-labeled peptides. Moreover, after five LC-SRM runs we verified the relevance of calibration by analyzing one of the calibration peptide solution at 10 fmole/µl.

The detection limit was defined as the lowest concentration determined on the linear part of calibration curve. It varies for different peptides in the range from 100 amole/µl to 200 amole/µl.

Labeled (SIS)/target peptide peak area ratios were used to calculate the concentration of the targeted peptide in a sample. Peak area ratios were obtained using Skyline software.

C_pept_ =C_lab_*S_pept_/S_lab_ where C_pept_ – target peptide concentration, C_lab_ – labeled peptide (SIS) concentration (see *Quantification*), S_pept_ – area of target peptide peak, and S_lab_ – area of labeled peptide peak.

All calculated target peptide copy numbers for liver tissue and HepG2 are listed in the Supplementary Table 1.

## Conflicts of interest

None

## Footnotes

Transparency document Transparency data associated with this article can be found in the online version at doi:10.1016/j.dib.2016.07.034.

## References

[1] Zgoda V.G., Kopylov A.T., Tikhonova O.V. (2013). Chromosome 18 transcriptome profiling and targeted proteome mapping in depleted plasma, liver tissue and HepG2 cells. J. Proteome Res..

[2] Ponomarenko E.A., Kopylov A.T., Lisitsa A.V. (2014). Chromosome 18 transcriptoproteome of liver tissue and HepG2 Cells and targeted proteome mapping in depleted plasma: update 2013. J. Proteome Res..

[3] Poverennaya E.V., Kopylov A.T., Ponomarenko E.A. (2016). State of the chromosome 18-centric HPP in 2016: transcriptome and proteome profiling of liver tissue and HepG2 cells. J. Proteom Res..

[4] Hood C.A., Fuentes G., Patel H., Page K., Menakuru M., Park J.H. (2008). Fast conventional Fmoc solid-phase peptide synthesis with HCTU. J. Pept. Sci..

[5] Fekkes D. (1996). State-of-the-art of high-performance liquid chromatographic analysis of amino acids in physiological samples. J. Chromatogr. B. Biomed. Appl..

[6] Ludwig C., Claassen M., Schmidt A., Aebersold R. (2012). Estimation of absolute protein quantities of unlabeled samples by selected reaction monitoring mass spectrometry. Mol. Cell. Proteom. [Internet].

[7] MacLean B., Tomazela D.M., Shulman N. (2010). Skyline: an open source document editor for creating and analyzing targeted proteomics experiments. Bioinformatics [Internet].

